# Clinical Characteristics and Survival Analysis of Patients with Supraclavicular Fossa Lymphadenopathy

**DOI:** 10.3390/diagnostics15121480

**Published:** 2025-06-11

**Authors:** Yi-Jou Kao, Wan-Lun Hsu, Yong-Chen Chen, Wu-Chia Lo, Ping-Chia Cheng, Li-Jen Liao

**Affiliations:** 1 School of Medicine, College of Medicine, Fu Jen Catholic University, New Taipei City 242062, Taiwan; girl510461@gmail.com; 2 Master Program of Big Data in Medical Healthcare Industry, College of Medicine, Fu Jen Catholic University, New Taipei City 242062, Taiwan; wanlun0112@gmail.com; 3 Data Science Center, College of Medicine, Fu Jen Catholic University, New Taipei City 242062, Taiwan; 4 Post-Baccalaureate Program in Nursing, Fu Jen Catholic University, New Taipei City 242062, Taiwan; 5 Department of Otolaryngology, Head and Neck Surgery, Far Eastern Memorial Hospital, New Taipei City 220216, Taiwan; lowuchia@gmail.com (W.-C.L.); i.cruising@gmail.com (P.-C.C.); 6 Head and Neck Cancer Surveillance & Research Group, Far Eastern Memorial Hospital, New Taipei City 220216, Taiwan; 7 Graduate Institute of Medicine, Yuan Ze University, Taoyuan 320315, Taiwan; 8 Department of Electrical Engineering, Yuan Ze University, Taoyuan 320315, Taiwan

**Keywords:** supraclavicular lymphadenopathy, malignancy, prognostic factors, overall survival, hematological parameters, sonography

## Abstract

**Background:** Supraclavicular lymph nodes (SCLNs) are often indicative of malignancy, but the effectiveness of ultrasound (US) and hematological parameters in their assessment and the prognosis of patients with malignant SCLNs need further study. **Methods**: We retrospectively reviewed 348 patients with SCLNs from July 2007 to June 2023, including patients over 18 years of age who underwent fine needle aspiration (FNA) or core needle biopsy (CNB). Our analysis focused on clinical characteristics, US features and hematological parameters to differentiate between benign and malignant SCLNs and to assess their prognostic value, especially in the Asian population. **Results**: The malignancy rate was 49%, with lung cancer (22%) and lymphoma (16%) being the most common. The malignant nodes were larger and had a greater short-to-long axis ratio, irregular margins, and abnormal vascular patterns (*p* < 0.01). The 5-year survival rate for patients with malignant SCLNs was 40%. Blood markers, such as the neutrophil-to-lymphocyte ratio (NLR), platelet-to-lymphocyte ratio (PLR), and systemic immune–inflammation index (SII) (SII ≥ 970), were significant prognostic factors for overall survival (OS). Compared with lymphoma patients, patients with malignancies of supraclavicular and infraclavicular origins had significantly worse OS. **Conclusions**: Our findings highlight the importance of ultrasound in evaluating SCLNs. Furthermore, hematological markers (NLR, PLR, and SII) and the origin of SCLNs have important prognostic value.

## 1. Introduction

Supraclavicular lymph nodes (SCLNs), particularly left-sided nodes, often referred to as Virchow’s nodes, were first described by the renowned pathologist Rudolf Virchow in association with gastric carcinoma metastasis and are predominantly involved in adenocarcinoma [1]. Following this description, Troisier expanded our understanding by noting that other abdominal neoplasms could also metastasize to these nodes, resulting in their hardening and enlargement—a phenomenon now recognized as Troisier’s sign [2]. Recent studies have demonstrated that the malignancy rate for SCLNs are approximately 51.9% [3] and 54.5% [4], respectively. In conclusion, supraclavicular lymph node enlargement indicates a greater incidence of malignancy.

Ultrasound (US) is widely recognized for its remarkable accuracy in assessing cervical lymph nodes, and sonographic characteristics (size, short-to-long axis ratio, shape, boundary and vascularity pattern) are useful in differentiating benign from malignant lesions [5]. However, its effectiveness in assessing SCLNs is unclear.

The etiology of supraclavicular lymphadenopathy is diverse and includes malignant conditions such as metastasis and lymphoma, as well as benign causes such as granulomatous inflammation and other nonspecific diagnoses [6,7]. Common primary sites of SCLN metastasis include the lungs, breasts, gastrointestinal tract, prostate, thyroid, and nasopharyngeal regions [6,7,8]. In Spain, 60% of SCLN cases are malignant, predominantly originating from lung cancer, followed by breast cancer (13.8%) [7]. In contrast, another study noted that both breast and lung cancers similarly contributed to approximately 8% of SCLN malignancies [6].

Given the lymphatic drainage pathways and gravitational flow to SCLNs, metastasis to these nodes is often linked to head and neck tumors. However, studies of head and neck tumors have not consistently demonstrated a propensity for metastasis in the supraclavicular region [8]. Although many studies have described the primary origin and pathology of supraclavicular lymph nodes [6,7], the origin of SCLN malignancy is unclear, and data on the nature of SCLN are sparse in Asia.

The survival rate of patients with malignant SCLNs requires further study, and it is still unclear whether SCLN metastasis is associated with a poor prognosis. In recent study patients with distal lymph node metastasis also exhibit a slightly higher 3-year overall survival rate (62.7%) compared with those with ipsilateral supraclavicular lymph nodes metastasis (53.5%), although the difference is not statistically significant; by contrast, patients with other distant metastases have the poorest 3-year overall survival rate (38.2%) [9]. In middle and lower thoracic esophageal squamous cell carcinoma, SCLN involvement is considered a prognostic marker for worse outcomes and should be classified as distant metastasis [10]. Various hematological parameters have been reported to be associated with the prognosis of patients with malignancies. In recent years, systemic inflammatory factors, such as the neutrophil-to-lymphocyte ratio (NLR) and systemic inflammation index (SII), have been reported as novel prognostic biomarkers for NPC and other cancers [11]. However, their utility in SCLNs is unclear.

The purpose of this study was to evaluate the US features, primary origin, clinical significance, and survival rate of patients with SCLNs.

## 2. Materials and Methods

### 2.1. Patients and Methods

The study protocol was approved by the Far East Memorial Hospital institutional review board (IRB: 112119-E). This was a retrospective follow-up study. We reviewed the data of 499 patients with level 4 and 5b neck masses from July 2007 to June 2023. Those aged ≥ 18 years with SCLNs and who underwent FNAC or CNB were included. The exclusion criteria were age < 18 years; no US-FNA (fine needle aspiration) or CNB (core needle biopsy) data; and other neck masses (*n* = 151), such as thyroid nodules, hematomas, lipomas, thyroglossal duct (TGD) cysts, epidermoid cysts, sebaceous cysts, neuromas, carbuncles, paraspinal masses, thoracic duct cysts, and schwannomas.

In the determination of the final diagnosis, pathology results were employed whenever accessible. In cases where pathology findings were unavailable, a collaborative approach was undertaken through a multidisciplinary team meeting involving experts from the pathology, radiology, and oncology departments. Patients who had negative cytopathology results were followed for a minimum of 6 months to verify that no malignancy had subsequently developed in these LNs. We divided the origins of the primary sites into four categories: lymphoma, unknown (the origin of the malignancy was unknown), infraclavicular (the origin of the malignancy originated from the infraclavicular region), and supraclavicular (the origin of the malignancy originated from the supraclavicular region).

Sonograms were performed with one high-resolution 7 to 18 MHz real-time linear-array transducer (Aplio MX, Toshiba, Tokyo, Japan). The detailed method was described in our previous study [5]. The methods used for US feature classification have been well described in previous studies [12]. We recorded patients’ age, sex, pathology of the tumor type (benign or malignant), laterality (left, right or bilateral), characteristic lymph node under ultrasound (size, short/long ratio, margin and vascularity pattern), and origin of the lymphadenopathy. We also evaluated hematological parameters, including the neutrophil-to-lymphocyte ratio (NLR), platelet-to-lymphocyte ratio (PLR), lymphocyte-to-monocyte ratio (LMR), and systemic immune–inflammation index (SII). The SII was calculated as follows: (platelet count × neutrophil count)/lymphocyte count. Lymph node stage was classified according to the principle of neck nodal metastasis from an unknown primary site (SQCCUP) [13].

### 2.2. Statistical Analysis

Categorical variables are expressed as numbers (percentages), continuous variables are expressed as mean values (±standard deviations; SDs), and follow-up times are expressed as medians (interquartile ranges; IQRs). Student’s *t* tests and chi-square tests were used to determine the differences in clinical parameters (i.e., age, sex, laterality, diameter of the short and long axes, S/L ratio, margin, and vascularity pattern) between benign and malignant lymph nodes, as appropriate.

We used receiver operating characteristic (ROC) analysis to calculate the area under the ROC curve (AUC) and then determined the optimal cutoff point values for hematological parameters according to overall survival (OS). We defined OS as the time from diagnosis to death from any cause or the time of the last patient follow-up. The potential effects of clinical variables on OS were investigated using KM plots with log-rank tests and univariate Cox proportional hazards models. Variables identified as statistically significant in the univariate Cox proportional hazards model were subsequently included in a multivariate Cox proportional hazards model for further assessment of their independent prognostic significance for overall survival. The hazard ratios (HRs) and 95% confidence intervals (95% CIs) were subsequently calculated according to univariate or multivariate Cox regression models. All the statistical tests used in this study were two-sided, and *p* values less than 0.05 were regarded as significantly different. We used STATA software, version 18.0 (Stata Corporation, College Station, TX, USA), for the data analysis.

## 3. Results

### 3.1. Main Results

From July 2007 to June 2023, a total of 348 cases with SCLNs were documented, comprising 178 benign cases and 170 malignant cases. Of these patients with malignant LNs, 170 underwent pathological examination (25 underwent lymphadenectomy and 21 underwent core needle biopsy of cervical LNS; 112 of these underwent biopsy at the primary site). After a collaborative discussion with a multidisciplinary team, 12 patients were also diagnosed as malignant. Another 151 patients with negative cytopathology results were followed for at least 6 months to verify the subsequent presence of malignancy in these LNs. A total of 27 of the patients underwent resection surgery. The general characteristics of the enrolled patients are presented in Table 1. The mean age of the patients was 51 years, with females accounting for 189 patients (54%) and males accounting for 159 patients (46%). The proportion of malignant cases was 49% (170/348). On ultrasonography, the mean size of the cervical lymph node was recorded as 1.25 cm on the short axis and 2.04 cm on the long axis. Among the malignancies, the lung was the most common primary site (22%), followed by lymphoma (16%). All the breast cancer patients were female, with a mean age of 51 years. The reactive pattern constituted the largest portion of benign lymph nodes (82%), with tuberculosis accounting for 10%; thirteen out of seventeen patients with tuberculosis were female.

### 3.2. Comparison Between Malignant and Benign Lesions

A comparison of demographic data and US features between malignant and benign supraclavicular lymph nodes is presented in Table 2. Significant differences were observed in age, sex, and the laterality of the lymph node between malignant and benign cases. Patients with malignant SCLNs were older (57.5 ± 13.9) than those with benign SCLNs (44.4 ± 17.4, *p* < 0.01). In addition, a greater proportion of males (55%) than females (43%) presented with malignant SCLNs. Compared with right-sided SCLNs (37%), bilateral SCLNs (75%) and left-sided SCLNs (52%) tended to be malignant.

On ultrasound, malignant lymph nodes were larger in both the short (1.64 ± 0.75 cm) and long axes (2.43 ± 1.18 cm) than benign nodes (short axis: 0.88 ± 0.49 cm, *p* < 0.01; long axis: 1.66 ± 0.98 cm, *p* < 0.01). Additionally, the short-to-long axis ratio (S/L ratio) was greater in malignant compared with benign cases (average of 0.72 ± 0.23 cm vs. 0.56 ± 0.23 cm, respectively). An irregular margin (72%) of the SCLN indicated a greater possibility of malignancy. In terms of the vascularity pattern, those nodules classified as ‘other’ types (80%) presented a greater tendency toward malignancy. Conversely, nodules with hilar vascularity (92%) were more frequently benign.

### 3.3. Survival Analysis

**Figure 1:**
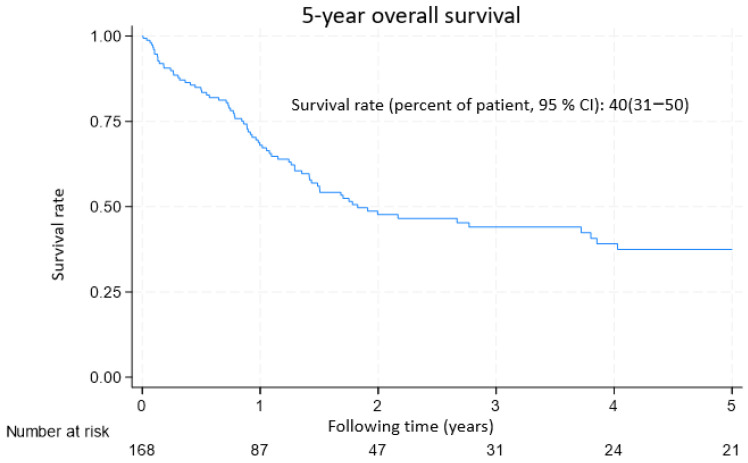
KM plot showing the overall survival of patients with malignant SCLNs.

For a median follow-up period of 2.0 years (interquartile range: 0.0–14.6 years), the 5-year survival rate was generally 40% (95% CI: 31–50%) for patients with malignant SCLNs (Figure 1).

**Figure 2:**
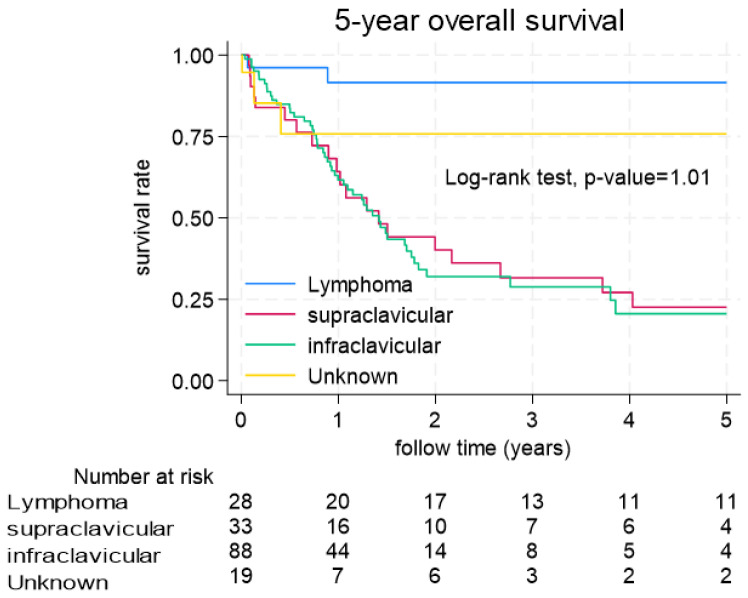
KM plots showing the survival analysis and stratification by primary site.

**Figure 3:**
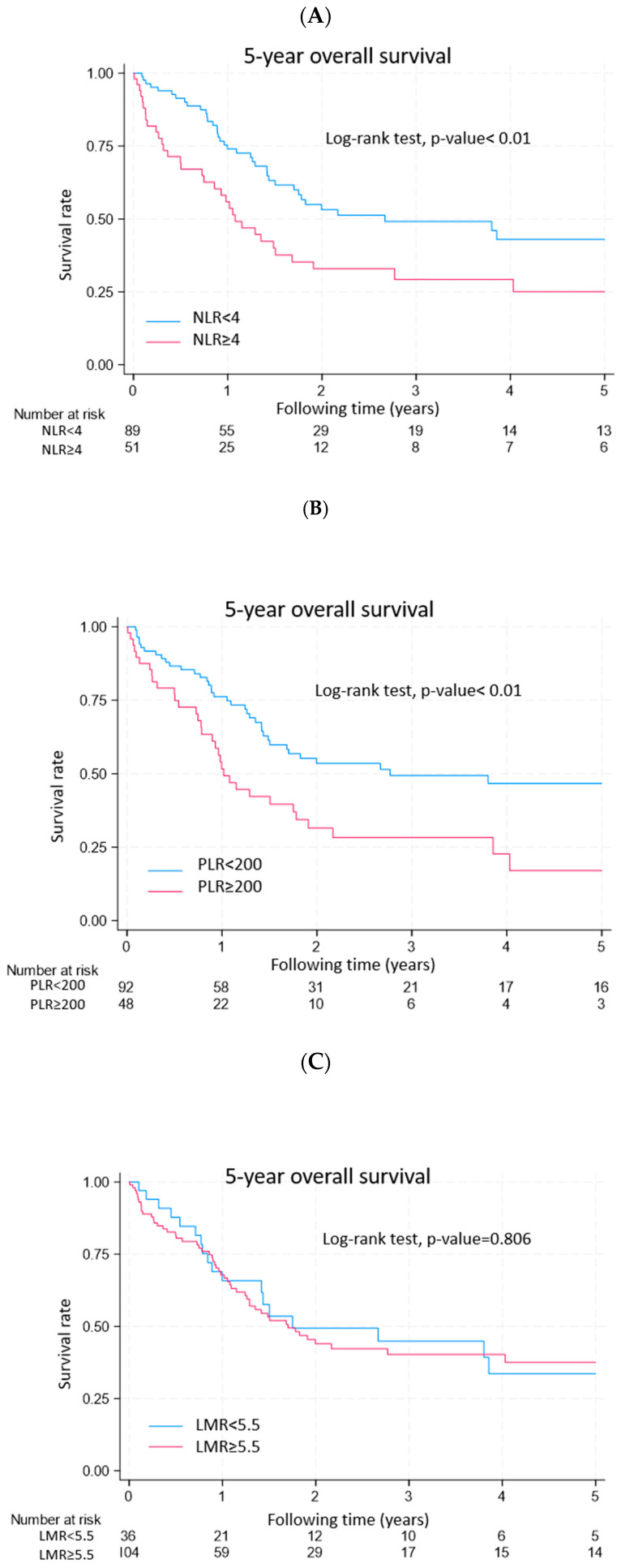

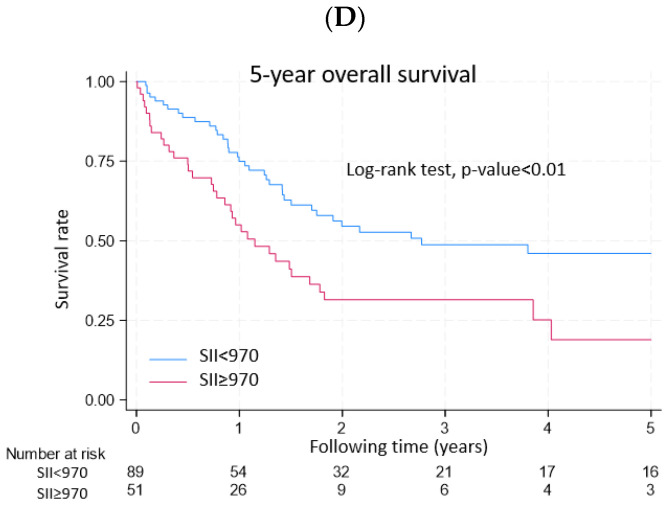
KM plots showing the survival analysis and stratification by different hematological parameters: (**A**) NLR, NLR < 4 versus NLR ≥ 4; (**B**) PLR, PLR < 200 versus PLR ≥ 200; (**C**) LMR, LMR < 5.5 versus LMR ≥ 5.5; (**D**) SII, SII < 970 versus SII ≥ 970.

Among the patients with malignancies, there was no significant difference in age, laterality, or stage. Compared with cancer originating from the supraclavicular and infraclavicular regions, lymphoma patients had a better prognosis (*p* = 0.101) (Figure 2). Hematological parameters such as NLR ≥ 4 (Figure 3A), PLR ≥ 200 (Figure 3B) and SII ≥ 970 (Figure 3D) reached statistical significance and were associated with a worse survival rate (log rank test < 0.01).

As shown in Table 3, multiple factors were related to OS in the univariate analysis, such as age (HR: 1.02, 95% CI: 1–1.02), primary site (unknown, HR: 2.02, 0.45–9.10; supraclavicular, HR: 7.25, 2.47–21.2 and infraclavicular, HR: 7.27, 2.60–20.3 compared with lymphoma), NLR (≥4, HR: 1.93, 1.22–3.04), PLR (≥200, HR: 1.39–3.5), and SII (≥970, HR: 2.18, 1.37–3.46).

In the multivariable analysis of OS (Table 3), only the SII was retained given the potential collinearity between the NLR, PLR, and SII. The primary site (unknown, HR: 1.93, 0.42–8.91; supraclavicular, HR: 6.26, 2.07–19.0; and infraclavicular, HR: 5.91, 2.07–16.90 compared with lymphoma) and the SII (≥970, HR: 1.92, 1.18–3.11) were found to be independent prognostic factors for OS.

### 3.4. Laterality of Various Origins of Malignant SCLNs

Comparisons of the laterality of various origins of malignant supraclavicular fossa lymphadenopathy are shown in Table 4. Gastrointestinal cancer (73%), urinary tract cancer (78%), and gynecology cancer (93%) tended to metastasize to the left side of the SCLNs. However, we still noted two of the eleven patients with gastrointestinal cancers and one of the nine patients with urinary tract cancers presented right-sided SCLNs. Each patient presented with bilateral SCLNs for gastrointestinal, urinary tract, and gynecological cancers.

## 4. Discussion

This study focused on the clinical characteristics and prognosis of a large series of SCLNs in an Asian population. The overall malignancy rate for SCLNs in our study was 49%, which is lower than that in previous reports [3,4]. We identified several factors significantly associated with malignancy, including age, US lymph node size, the S/R (short-to-long axis) ratio, irregular margins, and abnormal vascular patterns. Hematological parameters, particularly the systemic immune–inflammation index (SII), were determined to be significant independent prognostic factors for OS. Additionally, the origin site of the malignancy emerged as a crucial determinant of OS, and patients with cancers originating from the infraclavicular and supraclavicular regions had a poorer prognosis than those with lymphoma.

The malignancy rate in our study was 49%, which is slightly lower than some previously reported ranges and supports the consensus that SCLNs are frequently associated with malignancy. However, in our series, not all patients with benign SCLNs underwent US-FNA or CNB, and they may have been easily observed; thus, we may have even overestimated the malignancy rate.

The etiology of SCLNs may differ geographically, and lung cancer was the most common site in our study. A Spanish study reported a 60% malignancy rate in SCLNs, predominantly from lung cancer, which aligns with our findings that lung cancer is a leading primary source [7]. Smoking is a well-known factor associated with lung cancer. Compared to Europe and the USA, Asia has the highest incidence and mortality of lung cancer. However, in Taiwan, 66.3% of patients diagnosed with lung cancer did not have a smoking history, and their disease was likely related to a family history or genetic factors [14,15]. The second most common type of malignancy was lymphoma, which is different from the findings of other studies. Previous studies have reported variable findings regarding the second most common primary malignancy, which metastasizes in cervical lymph nodes. In a retrospective cohort, lung cancer accounted for the highest proportion (41.7%) of cases, followed by head and neck cancers (20.58%) and breast cancer (17.64%), indicating that head and neck malignancies were the second most common primary tumors in that population [16]. In a Spanish cytology-based series, lung cancer was again the most frequent primary site; however, the second most common origin varied depending on nodal laterality, with breast, gastrointestinal, or prostate cancers more frequently associated with left-sided SCLNs [7]. Meanwhile, an international review found that breast cancer was the most common distant primary (2.3–4.3%), followed by lung cancer (1.5–32%), making lung cancer the second most common overall in that global analysis [17]. In contrast to these findings, our study identified lymphoma (16%) as the second most frequent malignancy involving supraclavicular lymph nodes, following lung cancer (22%). These differences likely reflect regional patterns of disease prevalence and clinical practice, as well as variation in access to healthcare, and referral practices. This highlights the importance of population-specific data, such as that presented in our Asian study.

In addition to being the most common malignancy in our cohort, lung cancer is also the most anatomically and clinically relevant to supraclavicular lymph node (SCLN) involvement. Lymphatic drainage from the lungs passes through hilar and mediastinal nodes before reaching the supraclavicular region, especially the left SCLN via the thoracic duct. In contrast, axillary nodes primarily drain the breast, chest wall, and upper limbs, and are less commonly involved in thoracic malignancies.

Clinical studies support this anatomical pattern: supraclavicular node metastases were observed in 14.13% of lung cancer patients undergoing staging evaluation [18], whereas axillary lymph node involvement was reported in only 0.75% of patients, often in association with SCLN or systemic disease [19].

From a diagnostic perspective, the decision between biopsy and imaging when supraclavicular lymphadenopathy is observed depends on node palpability. For non-palpable SCLNs, Samson et al. demonstrated that ultrasound and contrast-enhanced CT-guided FNAC achieved high diagnostic accuracy in patients with suspected thoracic malignancies [20]. When FNAC is inconclusive or lymphoma is suspected, core needle or excisional biopsy should be considered to ensure sufficient tissue for subtyping. In contrast, in patients with palpable, clinically suspicious SCLNs, direct tissue sampling is typically preferred. The study emphasized that upfront biopsy may be more cost-effective than routine imaging in these cases. Furthermore, PET/CT is not recommended as an initial diagnostic tool unless contraindications to iodinated contrast exist [21].

A recent pooled analysis of data from seven high-income countries showed that men consistently had higher TB incidence rates than women, particularly in individuals over 15 years of age [22]. However, extrapulmonary tuberculosis is more common in women and young people, especially in the supraclavicular region. A previous study revealed a relatively high incidence of tuberculosis in benign cases due to the regional epidemiological pattern [23]. Although tuberculosis manifests primarily as a pulmonary disease, extrapulmonary presentations, known as extrapulmonary TB (EPTB), of the head and neck region are common in endemic countries [24]. Notably, most cases of EPTB are found in women. In this study, the reactive pattern constituted the largest portion of benign lymph nodes (82%), with tuberculosis accounting for 10%, reflecting the epidemiology of TB in Taiwan. In this study, thirteen out of seventeen patients (76%) with tuberculosis were women. According to previous reports, lymphadenopathy is the common form of EPTB, and the supraclavicular group (29.4%) was the most commonly enlarged lymph node in the cervical groups. Females represented 62% of the patients with enlarged supraclavicular lymph nodes [25]. A recent Indian study showed a female predominance (54.2%), attributed to biological and socioeconomic factors, with a notable incidence peak between 20 and 40 years due to enhanced immune responses facilitating granuloma detection [26]

Metastasis to SCLNs usually originates from primary tumors in the head and neck, breast or abdomen. Infradiaphragmatic tumors very rarely metastasize to these nodes [27]. Anatomically, it is expected that an infra-diaphragmatic malignancy would always metastasize to the left SCLNs through the thoracic duct. In our series, gastrointestinal cancer (73%), urinary tract cancer (78%), and gynecologic cancer (93%) metastasized to the left side of the SCLNs, and few patients showed metastasis to the right SCLNs. This pattern aligns with traditional anatomical expectations, although recent evidence suggests that infra-diaphragmatic malignancies may not exclusively involve the left SCLNs [4]. Furthermore, a systematic review revealed that esophageal cancer tends to metastasize to the right supraclavicular lymph node more often than to the left [28]. For right SCLNs, the differential diagnosis of infradiaphragmatic malignancies still needs to be considered.

US helps differentiate between benign and malignant lymph nodes, but no studies have focused on SCLN. In our study, malignant SCLNs tended to be larger than benign SCLNs. However, nodal size alone cannot reliably distinguish reactive from metastatic lymph nodes [29,30,31]. Our results are consistent with other studies showing that metastatic nodes are generally rounded, indicating that the short-to-long axis ratio (S/L ratio) is greater than 0.5 [18,29]. In addition, our study confirmed that an irregular margin of nodes had the highest predictability for malignancy [32]. Other studies have noted that hilar vascular patterns are thought to be suggestive of benign lesions [33], and our findings yielded similar results. These findings emphasize that US is also beneficial in the differential diagnosis of SCLNs.

Hematological parameters are prognostic factors in several cancers but have not been well established for SCLNs. Previous studies have shown that the NLR is an independent factor influencing the OS of patients with non-small cell lung cancer (NSCLC) [11]. Higher NLRs, PLRs, and SIIs are correlated with poorer OS and a greater risk of lymph node metastasis [34,35]. Our analysis revealed that several hematological parameters, such as the NLR, PLR, and SII, served as significant prognostic factors for patients with SCLN involvement. Notably, an elevated SII (≥970) was identified as an independent predictor of poor overall survival (OS) in our multivariate analysis (HR: 1.92, 95% CI: 1.18–3.11), highlighting its value as a simple and effective prognostic marker.

Different cancers have different lymph node staging criteria. To unify lymph node staging, we used SQCCUP [13] for malignant SCLN staging. SQCCUP is specifically designed for patients with an unknown primary site of the cervical lymph nodes. In our study, there was no difference in prognosis according to this nodal stage. Instead, simple classification according to lymphoma, supraclavicular origin, infraclavicular origin, and unknown primary origin was a better prognostic indicator for malignant SCLNs.

Although retrospective in design, this study provides novel insights by integrating ultrasound features, inflammatory markers, and primary tumor origin in patients with supraclavicular lymphadenopathy. Our findings highlight the diagnostic value of ultrasound and identify SII as an independent prognostic factor. Based on a large East Asian cohort, this study also offers region-specific data to guide future research and clinical practice.

While this study provides new insights into the diagnostic and prognostic evaluation of SCLNs, several limitations should be acknowledged. The retrospective design introduces inherent biases, and unnoticed or unavoidable selection bias nay have played a role in our findings. Additionally, practice patterns might vary among different institutions. Furthermore, the single-center nature of the study may limit its generalizability to other populations, particularly those outside of Asia. Furthermore, the exclusion of specific conditions and benign entities, although necessary for focus, may have implications for the comprehensiveness of our findings. In addition, we did not evaluate the correlation between baseline FDG-SUV values from PET-CT and ultrasonographic features (e.g., power-Doppler vascularity) of supraclavicular lymphadenopathies, as PET-CT was not routinely performed in all patients. The lack of standardized PET-CT imaging limits further exploration of metabolic–structural associations in lymph node characterization. Finally, the variation in cutoff values for prognostic markers, such as the SII, across different studies suggests that these thresholds may need adjustment on the basis of population-specific data. However, we tested various cutoff points, and the results consistently showed the same trend. In conclusion, multicenter studies or large-scale prospective trials are warranted to validate and potentially refine the prognostic markers identified in the present study.

## 5. Conclusions

Lung cancer and lymphoma were the main types of malignant SCLNs identified in this series. Ultrasonographic features—such as larger nodal size, higher short-to-long axis ratio, irregular margins, and non-hilar vascularity—were significantly more common in malignant than in benign nodes, underscoring the value of ultrasound in the initial diagnostic evaluation. In addition, hematological parameters (NLR, PLR, and SII) and the origin of the SCLNs demonstrated important prognostic significance.

## Figures and Tables

**Table 1:** Baseline characteristics of the study patients (*n* = 348).

Characteristic	*N* (%)/Mean ± SD	Characteristic	*N* (%)/Mean ± SD
Age, years	50.8 ± 17.1	Malignancy	
Sex		Lymphoma	28 (16)
Female	189 (54)	Unknown primary	21 (12)
Male	159 (46)	Supraclavicular origin	54 (32)
Final diagnosis		Head and neck cancer	20 (12)
Benign	178 (51)	Esophageal cancer	9 (5)
Malignancy	170 (49)	Thyroid cancer	3 (2)
Laterality		Thymus cancer	1 (1)
Left	156 (45)	Infraclavicular origin	88 (52)
Right	144 (41)	Lung cancer	37 (22)
Bilateral	48(14)	Gastrointestinal tract cancer	11 (6)
Size (cm)		Breast cancer	17 (10)
Short axis	1.25 ± 0.74	Gynecology cancer	13 (8)
Long axis	2.04 ± 1.15	General Urology tract cancer	9 (5)
Margin		Melanoma	1 (1)
Clear	242 (70)	Benign	
Vague	106 (30)	Reactive	146 (82)
Vascularity pattern		Tuberculosis	17 (10)
Avascular	123 (69)	Granulomatous and necrotizing lymphadenitis	15 (8)
Hilar	38 (11)		
Other	71 (20)		
Nodal stage			
N1	11 (6)		
N2 a	33 (19)		
N2 b	88 (52)		
N2 c	38 (22)		

**Table 2:** Comparisons of demographic data and US features between malignant and benign supraclavicular lymph nodes.

Variables	Malignancy	Benign	*p* Value
Age, years	57.5 ± 13.9	44.4 ± 17.4	<0.01
Sex			0.026
Female	82 (43)	107 (57)	
Male	88 (55)	71 (44)	
Laterality			<0.01
Left	81 (52)	75 (48)	
Right	53 (37)	91 (63)	
Bilateral	36 (75)	12 (25)	
Mean size, cm			<0.01
Short axis	1.64 ± 0.75	0.88 ± 0.49	
Long axis	2.43 ± 1.18	1.66 ± 0.98	
Short/Long axis ratio			<0.01
	0.72 ± 0.23	0.56 ± 0.23	
Margin			<0.01
Regular	94 (39)	148 (61)	
Irregular	76 (72)	30 (28)	
Vascularity pattern			<0.01
Avascular	110 (46)	129 (54)	
Hilar	3 (8)	35 (92)	
Other	57 (80)	14 (20)	

**Table 3:** Overall survival analysis of patients with malignant supraclavicular fossa lymphadenopathy with univariate and multivariate Cox regression.

	Univariate Cox Regression			Multivariate Cox Regression		
Variables	HR	95% CI	*p* Value	HR	95% CI	*p* Value
Age, years	1.02	(1.00–1.04)	0.021	1.02	(1.00–1.04)	0.074
Sex						
Female	Ref.					
Male	1.2	(0.77–1.87)	0.43	1.4	(0.84–2.35)	0.198
Laterality						
Right (*n* = 53)	Ref.					
Left (*n* = 81)	1.36	(0.78–2.38)	0.278			
Bilateral (*n* = 36)	1.87	(0.99–3.52)	0.053			
Nodal stage (SQCCUP)						
N1	Ref.			Ref.		
N2 a & N2 b	1.35	(0.49–3.75)	0.56	1.05	(0.37–3.02)	0.928
N2 c	1.77	(0.61–5.17)	0.295	1.46	(0.49–4.43)	0.5
Primary site						
Lymphoma (*n* = 28)	Ref.			Ref.		
Unknown (*n* = 21)	2.02	(0.45–9.1)	0.36	1.93	(0.42–8.91)	0.398
Supraclavicular (*n* = 33)	7.25	(2.47–21.2)	<0.01	5.91	(2.47–21.2)	<0.01
Infraclavicular (*n* = 88)	7.27	(2.6–20.3)	<0.01	6.26	(2.6–20.3)	<0.01
NLR						
<4	Ref.					
≥4	1.93	(1.22–3.4)	<0.01			
PLR						
<200	Ref.					
≥200	2.2	(1.39–3.5)	<0.01			
LMR						
≥5.5	Ref.					
<5.5	1.07	(0.64–1.79)	0.806			
SII						
<970	Ref.			Ref.		
≥970	2.18	(1.37–3.46)	<0.01	1.92	(1.18–3.11)	0.008

Abbreviations: neutrophil-to-lymphocyte ratio (NLR), platelet-to-lymphocyte ratio (PLR), lymphocyte-to-monocyte ratio (LMR), systemic immune–inflammation index (SII), squamous cervical cancer of unknown primary site (SQCCUP).

**Table 4:** Comparisons of the laterality of various origins of malignant supraclavicular fossa lymphadenopathy.

Cancer Origin	Bilateral *N* (%)	Left *N* (%)	Right *N* (%)	Total
Lung cancer	9 (24)	13 (35)	15 (41)	37 (22)
Lymphoma	9 (32)	10 (36)	9 (32)	28 (16)
Unknown primary	3 (14)	8 (38)	10 (48)	21 (12)
Breast cancer	4 (24)	9 (53)	4 (24)	17 (10)
Head and neck cancer	6 (30)	7 (35)	7 (35)	20 (12)
Esophageal cancer	2 (22)	4 (44)	3 (33)	9 (5)
Gastrointestinal tract cancer	1 (9)	8 (73)	2 (18)	11 (6)
Gynecology cancer	1 (7)	12 (93)	0 (0)	13 (8)
General Urology tract cancer	1 (11)	7 (78)	1 (11)	9 (5)
Thyroid cancer	0 (0)	2 (67)	1 (33)	3 (2)
Thymus cancer	0 (0)	1 (100)	0 (0)	1 (1)
Melanoma	0 (0)	0 (0)	1 (100)	1 (1)
Total	36	81	53	170

## Data Availability

The original contributions presented in this study are included in the article. Further inquiries can be directed to the corresponding author.

## Abbreviations

The following abbreviations are used in this manuscript:

SCLNs	Supraclavicular lymph nodes
US	Ultrasound
FNA	Fine needle aspiration
CNB	Core needle aspiration
NLR	Neutrophil-to-lymphocyte ratio
PLR	Platelet-to-lymphocyte ratio
LMR	Lymphocyte-to-monocyte ratio
SII	systemic immune–inflammation index
OS	Overall survival
NPC	Nasopharyngeal cancer
TGD	Thyroglossal duct
ROC	Receiver operating characteristic
AUC	Area under the ROC curve
95% CIs	95% confidence intervals
EPTB	Extrapulmonary tuberculosis
TB	Tuberculosis
KM curve	Kaplan–Meier curve
SQCCUP	Squamous cervical cancer of unknown primary site

## References

[1] Virchow R. (1848). Zur diagnose der krebse in unterleibe. Med. Reform..

[2] Troisier E. (1989). L’adenopathie sus-claviculaire dans les cancers ce l’abdomen. Arch. Gen. Med..

[3] Katta R., Chinnam A., Prasad U., Bora S. (2024). Fine-needle aspiration cytology of Virchow’s node: A 3-year experience in a tertiary care teaching hospital in South-Eastern belt of India. Indian J. Health Sci. Biomed. Res. KLEU.

[4] Qayoom S., Shabbir N., Sagar M., Jaiswal R., Akhtar N., Kumar M. (2025). Revisiting Virchow;s Node: Exploring the Diagnostic Spectrum of the Supraclavicular Lymph Node Through Fine-Needle Aspiration Cytology in a Tertiary Care Hospital. Turk Patoloji Derg..

[5] Liao L.J., Wang C.T., Young Y.H., Cheng P.W. (2010). Real-time and computerized sonographic scoring system for predicting malignant cervical lymphadenopathy. Head Neck.

[6] Gupta R.K., Naran S., Lallu S., Fauck R. (2003). The diagnostic value of fine needle aspiration cytology (FNAC) in the assessment of palpable supraclavicular lymph nodes: A study of 218 cases. Cytopathology.

[7] Fernández Aceñero M.J., Caso Viesca A., Díaz Del Arco C. (2019). Role of fine needle aspiration cytology in the management of supraclavicular lymph node metastasis: Review of our experience. Diagn. Cytopathol..

[8] Acar T., Savas R., Kocacelebi K., Guneyli S. (2014). Supraclavicular lymphadenopathy: Should it be perceived as the virchow’s node of head and neck tumors?. Oncol. Res. Treat..

[9] Pan H., Wang H., Qian M., Mao X., Shi G., Ma G., Yu M., Xie H., Ling L., Ding Q. (2021). Comparison of Survival Outcomes Among Patients With Breast Cancer With Distant vs Ipsilateral Supraclavicular Lymph Node Metastases. JAMA Netw Open.

[10] Wang F., Ge X., Wang Z., Weng Y., Yin R., You Q. (2020). Clinical significance and prognosis of supraclavicular lymph node metastasis in patients with thoracic esophageal cancer. Ann. Transl. Med..

[11] Erciyestepe M., Selvi O., Dinç Sonuşen Ş., Öztürk A.E., Dinç G., Güneş T.K., Aydın O., Yaşar N., Balkaya Aykut G., Vatansever S. (2024). Prognostic Value of Inflammation and Nutrition-Based Scores in Non-Small Cell Lung Cancer. Med. Princ. Pract..

[12] Luo J., Jin P., Chen J., Chen Y., Qiu F., Wang T., Zhang Y., Pan H., Hong Y., Huang P. (2023). Clinical features combined with ultrasound-based radiomics nomogram for discrimination between benign and malignant lesions in ultrasound suspected supraclavicular lymphadenectasis. Front. Oncol..

[13] Pavlidis N., Pentheroudakis G., Plataniotis G. (2009). Cervical lymph node metastases of squamous cell carcinoma from an unknown primary site: A favourable prognosis subset of patients with CUP. Clin. Transl. Oncol..

[14] Luo Y.H., Chiu C.H., Scott Kuo C.H., Chou T.Y., Yeh Y.C., Hsu H.S., Yen S.H., Wu Y.H., Yang J.C., Liao B.C. (2021). Lung Cancer in Republic of China. J. Thorac. Oncol..

[15] Lam D.C., Liam C.K., Andarini S., Park S., Tan D.S.W., Singh N., Jang S.H., Vardhanabhuti V., Ramos A.B., Nakayama T. (2023). Lung Cancer Screening in Asia: An Expert Consensus Report. J. Thorac. Oncol..

[16] Nagarkar R., Wagh A., Kokane G., Roy S., Vanjari S. (2019). Cervical Lymph Nodes: A Hotbed For Metastasis in Malignancy. Indian J. Otolaryngol. Head Neck Surg..

[17] Lopez F., Rodrigo J.P., Silver C.E., Haigentz M., Bishop J.A., Strojan P., Hartl D.M., Bradley P.J., Mendenhall W.M., Suarez C. (2016). Cervical lymph node metastases from remote primary tumor sites. Head Neck.

[18] Lamichhane S., Thapa A., Chataut D., Suwal S., Ansari M.A., Yadav B.K. (2023). Metastatic Supraclavicular Lymph Nodes among Patients with Lung Carcinoma in a Tertiary Care Centre: A Descriptive Cross-sectional Study. JNMA J. Nepal. Med. Assoc..

[19] Satoh H., Ishikawa H., Kagohashi K., Kurishima K., Sekizawa K. (2009). Axillary lymph node metastasis in lung cancer. Med. Oncol..

[20] Samson V.K.N., Adimulapu S., Gafoor J.A. (2025). A Clinical Trial on Ultrasound and Enhanced CT scan Guided FNAC of Non-Palpable Supra-Clavicular Lymph Nodes in Suspected Malignancies of the Lungs and Pleura. Int. J. Pharm. Clin. Res..

[21] Hanzalova I., Matter M. (2024). Peripheral lymphadenopathy of unknown origin in adults: A diagnostic approach emphasizing the malignancy hypothesis. Swiss Med. Wkly.

[22] Peer V., Schwartz N., Green M.S. (2022). Gender differences in tuberculosis incidence rates-A pooled analysis of data from seven high-income countries by age group and time period. Front Public Health.

[23] Chen S.C., Wang T.Y., Tsai H.C., Chen C.Y., Lu T.H., Lin Y.J., You S.H., Yang Y.F., Liao C.M. (2022). Demographic Control Measure Implications of Tuberculosis Infection for Migrant Workers across Taiwan Regions. Int. J. Environ. Res. Public Health.

[24] Tsai Z.L., Yong C.Y., Wu Y.C., Fang C.Y. (2022). Extrapulmonary tuberculosis in the head and neck-a rare situation deserves attention. J. Dent. Sci..

[25] Singh S.K., Tiwari K.K. (2016). Tuberculous lymphadenopathy: Experience from the referral center of Northern India. Niger. Med. J..

[26] Gupta A., Kunder S., Hazra D., Shenoy V.P., Chawla K. (2021). Tubercular lymphadenitis in the 21st century: A 5-Year single-center retrospective study from South India. Int. J. Mycobacteriol..

[27] Gupta A., Mirpuri L., Hassan H., Malik F., Amtul N. (2023). Ureteral transitional cell carcinoma with supraclavicular lymph node metastasis: A case report. J. Surg. Case Rep..

[28] Hagens E.R.C., van Berge Henegouwen M.I., Gisbertz S.S. (2020). Distribution of Lymph Node Metastases in Esophageal Carcinoma Patients Undergoing Upfront Surgery: A Systematic Review. Cancers.

[29] Ahuja A.T., Ying M., Ho S.Y., Antonio G., Lee Y.P., King A.D., Wong K.T. (2008). Ultrasound of malignant cervical lymph nodes. Cancer Imaging.

[30] Liu C., Xiao C., Chen J., Li X., Feng Z., Gao Q., Liu Z. (2019). Risk factor analysis for predicting cervical lymph node metastasis in papillary thyroid carcinoma: A study of 966 patients. BMC Cancer.

[31] Tong J., Lin T., Wen B., Chen P., Wang Y., Yu Y., Chen M., Yang G. (2023). The value of multimodal ultrasound in diagnosis of cervical lymphadenopathy: Can real-time elastography help identify benign and malignant lymph nodes?. Front Oncol..

[32] Jayapal N., Ram S.K.M., Murthy V.S., Basheer S.A., Shamsuddin S.V., Khan A.B. (2019). Differentiation Between Benign and Metastatic Cervical Lymph Nodes Using Ultrasound. J. Pharm. Bioallied. Sci..

[33] Wang B., Guo Q., Wang J.Y., Yu Y., Yi A.J., Cui X.W., Dietrich C.F. (2021). Ultrasound Elastography for the Evaluation of Lymph Nodes. Front Oncol..

[34] Ji Y., Wang H. (2020). Prognostic prediction of systemic immune-inflammation index for patients with gynecological and breast cancers: A meta-analysis. World J. Surg. Oncol..

[35] Li P., Li H., Ding S., Zhou J. (2022). NLR, PLR, LMR and MWR as diagnostic and prognostic markers for laryngeal carcinoma. Am. J. Transl. Res..

